# Bibliographical Notices

**Published:** 1854-04

**Authors:** 


### ART. IV.—BIBLIOGRAPHICAL NOTICES.

*History of the Epidemic Yellow Fever at New Orleans, La., in* 1853, by E. D. FENNER, M. D.

*Tableau of the Yellow Fever of* 1853, *with Topographical, Chronological and Historical Sketches of the Epidemics of New Orleans, since their origin in* 1796, *illustrative of the Quarantine Question*, by BENNET DOWLER, M. D.

Such are the titles of two very interesting papers, a synopsis of which we have thought might be acceptable to the readers of our Journal.

Dr. Fenner is an earnest and honest worker; his past and present labors clearly prove this; he not only works himself, but induces his professional friends, within the extensive range of his influence, to work with and for him. The paper now under consideration, is a plain and unostentatious narrative of the facts and circumstances attending the epidemic of 1853; these facts and circumstances have evidently been collected with uncommon care and industry, and are set forth with a simplicity, candor and force well calculated to make an effective impression upon both the professional and general reader. Such is the purpose of Dr. Fenner. The Quarantine question seems yet to be a mooted one with the people, and to some extent with the profession of New Orleans; the history of Dr. Fenner, though altogether truthful and impartial, is strongly opposed to all quarantine regulations, and is so fashioned as to lead to a like conviction in the minds of his readers.

The *Tableau* of Dr. Dowler is a more elaborate production, more ambitious in style, more ostentatious of learning, evidently designed by its marginal index as a work of reference for future historians and students of Yellow Fever, not limited to the epidemic of 1853, but sketching all the epidemics which have visited New Orleans since 1796, and epidemics generally; it is equally hostile with the paper of Dr. Fenner, to all quarantine regulations, which are vigorously assailed by the triple battery of fact, reasoning and ridicule.

After some very just prefatory remarks upon the culpable indifference manifested by the people and city authorities of New Orleans, in regard to the local conditions favoring the development of Yellow Fever, during the existence of an epidemic influence, the history of Dr. Fenner is arranged into a number of separate parts, which in the aggregate comprehend the entire subject.

The first division has reference to the meteorology of New Orleans, a branch of the subject of great interest in the estimation of many, in regard to the etiology of Yellow Fever. Dr. Fenner procured his meteorological tables from the register of Dr. E. H. Barton, a member of the Sanitary Commission and very accurate in his observations; these extend through all the months of 1853

up to October. The calculations are given in detail, and lead to the following general conclusions on the part of Dr. Fenner.

"The winter was quite cool and dry, but without any very cold weather. I did not see ice in the open gutters of the city, nor was the ground frozen in the heart of the city."

"The spring was quite variable; alternately hot and cold, and rather dry. Vegetation *remarkably backward.* From the 12th to the 20th of May, the days were *very hot*, whilst the nights were so cold as to render one or two blankets necessary to comfort. The thermometer rose to 88° which is within 1° of the highest point in July. On the 21st there was a sudden change, the weather becoming quite cold. This continued for two or three days; and it was about this time, as well as I have been able to ascertain, that the few cases of Yellow Fever appeared in the city."

"The summer was hot, though not unusually so. It will be seen that the *average* of the thermometer was above 79°, which some contend is necessary for the prevalence of yellow fever as an epidemic. The summer was likewise *quite wet*, though not as much so as others in which there was not so much Yellow Fever."

The meteorological tableau of Dr. Dowler contains some very remarkable facts interesting to those who recognize a high temperature as one of the most important of the appreciable conditions of yellow fever.

"The most remarkable feature in the weather of the summer of 1853 is that of the diminished heat in the whole tier of Southern States bordering on the Gulf of Mexico, in which yellow fever prevailed, compared with the Western, Middle and North-eastern portions of the Republic. L. Blodget, Esq., in charge of the Meteorological Department of the Smithsonian Institution, in an able paper, 'on the climatic conditions of the summer of 1853, most directly affecting its sanitary character,' an official report for June, July and August, from ninety meteorological stations from Canada to Florida and Texas, shows that in the fourth week of June, the maximum heat from New York to Savannah, gave an average of 95°. Now the corresponding week in New Orleans gave, according to Lillie's tables for the corresponding week, only 92° as the mean maximum, and throughout the whole epidemic, the average, never for any week, equaled that of the above mentioned central zone, where no yellow fever appeared. The average maximum temperature of the week ending August 26th, in New Orleans, was 91°, while the mortality was the greatest, amounting to 1.667, or more than 238, as the average per day, the temperature averaging one degree more than that of the week ending September the 15th, during which the mortality was only 411, averaging less than 59 per day. The average maximum temperature of the week ending October the 22d was 82°, two degrees more than that of the preceding week, though the number of deaths did not differ more than three for that period."

"It was not until June that the yellow fever showed itself even in the sporadic form to auy considerable degree—the week ending on the 30th of the month, gave as the average of maxima 92° in New Orleans. Now, Mr. Blodget's tables will show that on and about the day aforesaid, the maxima temperatures were as follows at the places indicated where yellow fever did not show itself:—Alexandria, Virginia, 95°; Knoxville, Tennessee, 94.4°; Oberlin, Ohio, 95°, 17th 97°; Baltimore, 92.3°, 22nd 96.2°; Camden, South Carolina, 97.6°; Sparta, Georgia, 97°; Eutaw, Alabama, 101°, (the day before 104°); Lebanon, Tennessee, 95.90°; New Harmony, Indiana, 28th, 97.5°; Bloomfield, New York, 21st, 95.5°; Philadelphia, 95°, 20th, 97°; Brooklyn, Michigan, 21st, 97°; Pouthney, Iowa, 20th, 97°. On the other hand, many Southern towns were comparatively cool—those which escaped, as well as those which suffered from yellow fever. Jacksonville, Florida, on the last day of June, 84°, Pensacola, 85°. Again, compare the 15th of August,—Smithsonian Institution, 91°, (the 14th 64°,); Alexandria, Virginia, 82.3°; Culloden, Georgia, 82.40°; Austin, Texas, 82°. This period, including the preceding two weeks and the week succeeding, was the hottest part of the season in New Orleans, the maximum ranging from 93° to 94°, being much greater than that which attended the invasion of the epidemic. The week ending the 28th of July, gave an average of 87°, although the mortality at that time, from yellow fever, fell but little short of 1400 during the month."

"If we compare the summer heat, (June, July and August,) of the yellow fever zone, with Northern latitudes, where yellow fever did not appear, it will be found that even the mean temperatures of the entire hot season correspond very nearly in many instances; the mean of New Harmony, Indiana, for June, 78.3°, nearly the same as Pensacola, which is 80°; Baltimore, 77.7°; Savannah, 79°; Lebanon, Ky., 79.5°; Camden, S. C., 79.3°; Danville, Ky., 79.3°; Mount Vernon, Ohio, 78.9°, agreeing within an inconsiderable fraction with Cedar Keys, Talahassee, Pensacola, and Jacksonville, (78.9°) in Florida, and Eutaw, Ala., Austin, Texas, and other places."

"The quantity of rain which fell in New Orleans in July, August and September, amounted to 16.81 inches, nearly two-thirds of which fell in July, which is usually the most rainy month in New Orleans, nearly one-third fell in the next month, leaving but the fraction of an inch for the latter, which, with the month of October, is the dryest season of the year."

"On comparing July and August, the two great epidemic months in New Orleans in 1853, it will be seen that there was nothing peculiar—nothing that can account for the epidemic in regard to the quantity of rain, which was in some places greater or less than in regions free from the fever, and sometimes similar. In these two months there fell 16.81 inches of rain in New Orleans; at West Point, N. Y., 18.28; at Richmond, Mass., 14.235; at Montreal, 10.191; at Philadelphia, 9.37; at Richmond, Va., 8.63; at Ann Harbor, Michigan, 5.06; at Bedford, Pa., 3.877; at Sanannah, 14.682; at Jacksonville, Florida, 10.1; at Pensacola, 4.078; at Cedar Keys, Florida, 15.1; Eutaw, Alabama, 14.739, &c."

From these facts, in regard to which Drs. FENNER and DOWLER for the most part concur, it will be seen that there was nothing very peculiar in the meteorological condition of New Orleans explanatory of the epidemic of 1853, nothing certainly to account for its unusual malignity; the cause of both must be sought in other and possibly more obscure conditions. While Dr. FENNER states in general terms that the season was a hot one, he also states that it was not distinguished by this feature from other seasons which were exempt from epidemic yellow fever. From Dr. DOWLER we learn that instead of excessive temperature, the most remarkable characteristic of the season was the *diminished* heat in all the Southern States bordering on the Gulf, in which the yellow fever prevailed.

From both historians we learn that there was nothing notable in the amount of rain; neither an excess nor a deficiency; nothing which had not been observed during seasons when yellow fever did not appear. Dr. DOWLER dwells somewhat specially, and in detail, upon this point, for the reason that "writers often most pertinaciously argue that yellow fever is owing to rain, when a rainy season and an epidemic happen together, as in 1839, when during an epidemic, it rained almost every afternoon for nearly two months. On the other hand, when an unusual drought and a severe epidemic prevailed in 1837, it was argued in like manner, that the absence of rain for a like period, (with the exception of a few showers) was the source of the evil."

The second division of Dr. FENNER's history is devoted to the sanitary condition of the city. According to Dr. FENNER, when the yellow fever appeared in New Orleans in 1853, the sanitary condition of all parts of the city was worse than he had ever known it to be; this statement is founded upon personal observation and is substantiated by the testimony of the different newspapers of the day, which earnestly invoked the attention of the public authorities and of the citizens to the subject. Dr. FENNER remarks, "that when this pestilence broke out, the condition of the city in respect to *cleanliness* was so bad as to be an object of public notoriety. Indeed, it was in such a state, that if it had given rise to Egyptian Plague instead of yellow fever, it should not have surprised any one. The only surprising thing is, that with so much filth of all kinds as is always to be found in this city, we do not have an epidemic every year. The fact that *we do not*, has even led to the public expression of the strange opinion that the public filth of the city, instead of *originating* yellow fever, absolutely *protects* us from it in no small degree."

Dr. FENNER has long been a most efficient advocate of sanitary reform in New Orleans; he considers the bad sanitary condition of the city, as one of the most powerful of the localizing causes of the various forms of fever more or less constantly prevailing there; he confidently anticipates a very great mitigation of all of them, and possibly the extermination of some of them, by an efficient system of sanitary measures. In reference to yellow fever he says, "time was when this disease was unknown here. From the best information I can obtain, New Orleans was in existence *three-quarters of a century* before the first epidemic of this disease prevailed here, and it is only within the present year that it has prevailed over the large plantations within sight of the city. Is it not possible to restore the sanitary condition that existed before this disease prevailed here? Many cities and towns have been severely scourged by yellow fever for successive years, but finally got rid of it."

In connection with his strictures upon the bad sanitary condition of New Orleans, Dr. FENNER takes occasion to administer a just rebuke to the editors of the daily journals, who, for the purpose of temporarily promoting the commercial interests of the city, studiously endeavor to conceal or suppress the true state of the health of the place, and thus induce many persons to remain until they are exposed to imminent danger for want of correct information.

Dr. Fenner comments more particularly upon the condition of streets, alleys and vacant lots, the exterior and more palpable sanitary defects of the city. Dr. Dowler, in his Sanitary Tableau, while concurring with Dr. Fenner in regard to these, is more special in his condemnation of the style of building in New Orleans; how this "has so long escaped the legislator, the grand jury, the landlord and the sanitarian, is marvellous. About ninety in every hundred houses, even in the richer portion of the city, are constructed in a manner that must be condemned in any climate, but in none so much as in this city, depressed as it is below the high water mark of the river everywhere, and in the rear, nearly on the sea level. The lower floor, which rots about four times in ten years, in a great majority of the houses, especially the stores, rests on the humid soil, sometimes at a lower level than the streets, no air being admitted underneath."

"Physicians in visiting the poor, especially in depressed portions of the city, must have often found the flooring of houses floating, and sometimes, after rains, quite covered with a water too filthy and offensive for description—laboratories for generating carbonic and other deadly gases, predisposing to disease and rendering recovery from any kind of sickness tedious, too often impossible."

In this connection, Dr. Dowler offers some advisory suggestions as to the best method of draining the city; he considers "effectual underground drainage scarcely a physical possibility in New Orleans—if possible, the expense could hardly be paid by the treasury of the United States, and if accomplished it would prove to be an intolerable nuisance." The position of New Orleans is such as not to afford soil elevation and descent sufficient for the successful working of subterranean sewers. "One heavy rain in New Orleans would change this abstract theory into a concrete mass of filth, which would fill these subterranean canals, which, lying as they must, below the level of the river, and even below the level of the sea and lake, would send forth emanations, strong, but not wholesome."

According to Dr. Dowler, the only feasible mode of draining New Orleans is by a well-devised plan of surface drainage; he thinks that "by adopting the experimental instead of the verbal or paper method of drainage, and using a moderate amount of common sense, much digging, and a good deal of capital, just as the Dutch have done in Holland, by which the land has been wrested from the dominion of the sea, New Orleans and its environs as far as Lake Pontchartrain, might be changed from mosquito lands and putrifying sheets of water, to horticultural, pasturage and meadow lands." Dr. Dowler prefers surface to underground drainage for all cities; while the position of New Orleans forbids the successful application of any other system, the object aimed at is more likely to be efficiently accomplished by surface drainage, even in those cities whose position is altogether favorable to underground drainage. "A gentlemen recently from Paris, and perhaps, the ablest quarantinist in New Orleans, informs me that in Paris underground drainage, with soil, elevation and declivity so vastly superior to New Orleans for this purpose, is mischievous. The Parisians find that the filth of the city accumulates in these subterranean sewers so as to send forth the most offensive and deleterious emanations. Hence, they prefer, after costly experiments, surface drainage, and wash off the filth into the Seine." The experience of many other cities accords with that of Paris; the whole argument in favor of surface drainage is briefly stated by the gentleman from Paris.

The commencement of the epidemic is dated by Dr. Fenner as early as the 23d of May; he observed the first case of *black vomit* on the 28th of May, in the wards of the Charity Hospital. This date is much earlier than that of other epidemics which have visited New Orleans.

The first cases were among the crew of the ship "Augusta," a vessel which arrived on the 17th of May, *direct from Bremen.* The fact that the disease originated among the shipping was calculated to suggest the suspicion that it was imported *from abroad*, and to the elucidation of this very important point, important in reference to future action upon quarantine measures, Dr. Fenner addressed himself with great patience and industry. He at once commenced a scrutinizing investigation, knowing how important it was to ascertain then all the facts and circumstances, and not to trust to a doubtful search for them after the lapse of even a few months. He learned that the "Augusta" was out from Bremen fifty-two days, and passed on the South of Cuba, not approaching nearer than thirty-five or forty miles of that island. She brought over two hundred and thirty European emigrants, who enjoyed good health on the voyage, having only lost two children of diarrhœa. The emigrants remained but one day in New Orleans, and then proceeded up to the West.

The "Camboden Castle," direct from Kingston, Jamaica, where the yellow fever was prevailing, was towed up from the mouth of the river in company with the "Augusta." At Kingston the "Camboden Castle" lost her captain with delerium tremens, and seven of her crew with yellow fever. *Not a single case of yellow fever* occurred on her during her passage from Kingston to New Orleans, nor while she remained in this latter port, up to the 8th of June. This fact is interesting as bearing upon the póssibility that the cases upon the "Augusta" might have been due to the free communication which took place between these vessels from the Balize to New Orleans; the entire exemption of the "Camboden Castle" from yellow fever, however, precludes this supposition.

The first case of yellow fever observed by Dr. Fenner, the one already referred to, was a passenger on the ship "Northampton," which arrived on the 10th of May, from Liverpool, passing fifty miles to the north of Cuba. This case left the ship upon her arrival, with the other passengers, and was not attacked until the 23d. The captain states that during his entire stay in New Orleans he had but one case of fever on board his ship, and that was a boy who was attacked on the 10th of June and recovered. After sailing on the 14th of June, the mate and captain were seized with the fever. All the facts of these cases and particularly of the first, are opposed to the supposition that the fever was imported by the "Northampton," or originated in any cause connected with that ship.

A number of other vessels are cited by Dr. Fenner, upon which or in the vicinity of which, cases of yellow fever occurred, but in every instance, he ascertained very clearly that they were free from this form of disease before their arrival, and that most of them were from ports in which yellow fever did not exist.

The most searching and important inquiry satisfied Dr. Fenner that the importation of the disease, by any of these vessels, was not true; he remarks, "it is often referred to as *a fact* that the first cases of yellow fever in this city *always occur among the shipping.* This is *not true*, as I have shown in my previous accounts of yellow fever in this city. In short, it is evident from the facts within my knowledge that the *shipping* has suffered much worse than the city from the contact."

From the shipping, Dr. Fenner extended his investigations to other localities in which the earlier cases occurred. By a comparison of dates, he found that the disease sprung up almost at the same time, in no less than *ten different places*, and these wholly disconnected with each other, and most of them so far removed from the vessels in which the very first cases occurred, as to forbid the supposition of intercommunication. "The localities designated, circumscribe *almost the entire outskirts of the city*, at the same time *dipping pretty far in.* Now, if any one can trace any sort of connection or communication between the first cases of the disease as they appeared in the different localities I have pointed out, or any thing like the *gradual* spread of an *imported contagion or infection from one or more points to the region around*, I can only say, it is more than I have been able, satisfactorily, to do."

It was currently reported that the yellow fever of 1853 was imported into New Orleans by vessels from Rio Janeiro. Dr. Fenner ascertained from unquestionable and authoritative sources that "nearly all the vessels that arrived from Rio, after the 1st of April, had suffered more or less from yellow fever, after leaving that port, but that *none of them had brought cases to New Orleans.*" Special mention is made of a number of these vessels, some of which arrived *long after* the disease had appeared in New Orleans. The ship "Adelaide" was reported to have brought the first cases; "upon careful inquiry," reports Dr. Fenner, "I find that this ship has not been here this year at all." The ship "Evangeline," against which the same accusation was made, did not arrive until the 10th of June, after a number of cases are known to have occurred. It is a singular circumstance that the people of Rio reciprocate the charge and allege that the fever was introduced among them by a vessel from Philadelphia. A writer in the New York Commercial Advertiser, in an account of the yellow fever at Rio

Janeiro, says that "the same fever pervaded the whole Brazilian coast in 1850. At Bahia, it was traced to a vessel from New Orleans, and believed by many to have been imported in her. With equal propriety it might have been said to have been imported into Rio from Philadelphia. Both would be absurdities, as the vessels left the United States in the winter season, and never had any sickness on board, until after their arrival." Dr. Fenner has clearly and fairly made out the same case in regard to the supposed introduction of the fever, by vessels arriving in New Orleans in 1853.

A fact of great interest, in its sanitary relations, is established by Dr. Fenner's inquiries into the localities in which yellow fever first most extensively and severely prevailed. He remarks, that "it is worthy of particular notice that the *epidemic provailed in the unpaved, and of course, least improved parts of the city all around, long before it did in the central and best improved parts.*" "I am not prepared," he further observes, "to maintain that the putrifying filth which is to be found to a great extent in this city at all times, is *the sole cause* of yellow fever; for if it were, we should certainly have an epidemic every year; but I do believe it is almost a *sine qua non*, and that if it were effectually removed, and the whole city well paved, it would effect more towards *the prevention* of yellow fever here than any thing that can be devised."

In the disposition manifested so constantly by yellow fever to display itself primarily and most severely in the outskirts and unimproved portions of a city, we perceive a strong analogy to the course ordinarily pursued by intermittent and remittent fevers and other forms of disease recognized as of miasmatic origin; the favorite seats of these are precisely the same localities, and they steadily recede and ultimately fade away before the advancing improvements of a city. Dr. Fenner thinks the *fever-cause* of yellow, remittent, and intermittent fever identical in all, producing the different types by merely a modification of its intensity. "The general remote cause of all the endemic fevers seen here, which, in its most virulent or powerful state, produces an epidemic of yellow fever, gradually undergoes modification in the progress of time; it becomes less virulent or powerful, and in proportion to the degree of this change, will it produce the milder types of fever. This position is illustrated by the remark of a highly intelligent practitioner of the fourth district, whom I met about the 10th of October. On being asked whether he saw much of yellow fever at that time, he replied "that he saw a good deal of fever, but he hardly knew whether it was *yellow fever* or not." I asked him how the cases looked, if they became *dangerously ill or died?* He said, "they then generally looked precisely like yellow fever." Another professional friend in the same district told me a short time afterwards that he had just been astonished at seeing a case of intermittent fever terminate in *fatal black vomit.* Such observations show that the prevailing *fever cause*, which in August produced *yellow fever* in nine-tenths of the cases that occurred, had become so much weakened in October as to produce mostly the remittent and intermittent types; yet the relationship between them was still so close, that if the latter were not promptly cured, they ultimately presented the worst features of the former."

Dr. Dowler, in his Geographical Tableau, opposes the opinion that marsh-miasma is concerned in the causation of yellow fever, and adduces a number of pertinent illustrations afforded by the epidemic of 1853, adverse to this doctrine. On this point it may be interesting to quote rather fully from the Tableau. "As immense importance has always been attached to the topography of yellow fever, which has been generally attributed to swamp-exhalation, it will be necessary to take a closer view."

"The elevated zone called the bluffs, a broken diluvial plateau, touching the Lakes Pontchartrain and Maurepas on the South, where it is most depressed, running North between the Pearl and Mississippi rivers; the Eastern shore of the latter for hundreds of miles, with some interruptions, is overlooked by these impending terraces, which sustain forests of colossal magnolias, pines, oaks, liquidambars, &c.,—a platform which sundry learned medical writers have indicated as a secure retreat from yellow fever, although neither the past nor the present justify this theoretical view. The epidemic of 1853 raged fully as much in this region as in the most depressed plains among the vast cypress swamps and salt-water marshes of littoral Louisiana. The epidemic was most fatal in this region, from its Southern border upon the Northern shore of Lake Pontchartrain at Madisonville, Mandeville,

Louisburg and Covington, to the higher lands of Baton Rouge, Clinton, Port Hudson, Jackson, Bayou Sara, St. Francisville, Fort Adams, Natchez, Grand Gulf, Yazoo and Vicksburg, not sparing the little villages of the piny forests."

"Thus the towns of Louisiana, Alabama, and Mississippi, States elevated from twenty to four hundred feet, and more, situated on the tertiary formation, often in the pine lands, remote from swamps, being high, dry and clean, suffered more, in many instances, than New Orleans situated, as it is, upon the recent alluvium or newer pliocene, touching the river in front and dipping into the stagnant swamps of the cypress basin in the rear, and intersected everywhere with filthy gutters, sewers, ditches or canals. The elevated zone of pine woods in Northern Louisiana, and elsewhere in the adjoining States, forms a striking contrast to the depressed plains, cypress basins, and marshes of the Southern delta. The epidemic of 1853, like previous ones, goes to prove that marsh miasma is not the specific cause of yellow fever, as is generally supposed. The very towns which the lamented DRAKE recently designated, on theoretical grounds, as safe retreats from yellow fever, have suffered most from it."

After citing a number of towns in which yellow fever appeared in 1853, some in elevated and some in depressed situations, Dr. DOWLER concludes his topographical sketch with the deduction "that it is evident that *altitude* did not modify the epidemic of 1853. The general opinion that yellow fever appears only in depressed places, or marshy plains, is contradicted by innumerable facts in America and Europe."

Dr. DOWLER, whilst denying to marsh miasma the position of a generating cause of yellow fever, offers no substitute; he thinks that the present state of medical science cannot offer any satisfactory explanation of the "*Ens Epidemicum.*" "A humiliating but true confession this. Heat, rain, moisture, swamps, *vegeto-animal* decomposition, contagion and numerous other alleged causes are unsatisfactory and inadequate, as might be shown by travelling over hundreds of inconclusive and contradictory volumes filled with special pleadings, diluted logic, theoretical biases and irrelevant facts.

"It is most certainly the duty of every writer on yellow fever to explain the cause of it if he can, but it is equally his duty not to sin against the decalogue of logic, any more than against the decalogue of Moses. Fortunately, the *conditions* if not the *causes* of yellow fever are to a considerable extent known; for example, it is known to be connected, no matter how, with the warm season of the year, with unacclimated constitutions, with aggregations of people in towns and villages, etc. It rarely attacks rural populations unless they crowd together so as to become virtually towns."

"A correct appreciation of these conditions is next in importance to the discovery of the cause of yellow fever—probably, the former may prove after all to be more important: for the discovery of the cause by no means warrants the conclusion that it is necessarily a removeable or a remediable one."

The yellow fever of 1853, though existing in a sporadic form for a longer time, prevailed as an epidemic only *two months.* Dr. Fenner says, "this again corresponds with the observations of the past. We have more or less yellow fever *every year*; but after a constant residence here of twelve years, I must say, that I have never known it prevail *to an epidemical extent* for a longer period than *sixty or seventy days.*"

Dr. Fenner denies the correctness of the prevalent popular opinion. "*that an epidemic can alone be extinguished by frost.*" He says that he demonstrated this error in his histories of the epidemics of 1847 and '48. "In 1847 the epidemic ceased long before the appearance of frost; yet sporadic cases were seen to a considerable extent to the end of the year. On the present occasion, the disappearance of the epidemic was published by the Board of Health on the 13th of October, whilst the *first frost* was announced on the 25th of October and that was only observed on the outskirts of the city. On the 31st there was a much heavier frost, but by this time, a large number of persons had returned to the city. It is true that some of them were attacked with the fever as before stated, but the cases were generally mild and easily managed."

Whilst the histories of Dr. Fenner prove that yellow fever often ceases to exist as an epidemic before the appearance of frost, the history of Dr. Dowler presents several instances in which the epidemic continued to progress unmodified by the occurrence of frost. "About the 25th of October a white frost appeared, for a few nights, at many of the interior towns of Louisiana, which was received as the harbinger of returning health, but which did not, in a marked degree, arrest the march of the epidemic. Warm weather, however, soon returned, and has continued to the present

(the third week of December,) but this did not revive the epidemic in places where it had declined, as in New Orleans and in many other places." "In the town of Clinton, in the Parish of West Feliciana, lying between the Mississippi and Pearl rivers, one hundred miles Northwest from New Orleans, the epidemic began about one month before this frost, but at the latest dates, (Dec. 10) it had not yet disappeared, seventy-five having died out of three hundred and fifty or four hundred who did not fly from the town, as did about one thousand persons."

Our notice of the papers of Drs. Fenner and Dowler has extended thus far, without comprehending more than a moiety of the valuable facts which they contain. We hope to complete our abstract in a future number. L. R.

